# Prognostic value of neutrophil count to albumin ratio in patients with decompensated cirrhosis

**DOI:** 10.1038/s41598-023-44842-9

**Published:** 2023-11-25

**Authors:** Junjie Yao, Xianbin Xu, Kai Gong, Huilan Tu, Zhaoyu Xu, Shaoheng Ye, Xia Yu, Yan Lan, Haoda Weng, Yu Shi

**Affiliations:** 1 State Key Laboratory for Diagnosis and Treatment of Infectious Diseases, National Clinical Research Center for Infectious Diseases, Collaborative Innovation Center for Diagnosis and Treatment of Infectious Diseases, The First Affiliated Hospital, Zhejiang University School of Medicine, Hangzhou, 310000 China; 2 Bethune Third Clinical Medical College, Jilin University, Changchun, 132000 Jilin China

**Keywords:** Biomarkers, Diseases, Risk factors

## Abstract

Our study aimed to investigate the prognostic value of neutrophil count to albumin ratio (NAR) in predicting short-term mortality of patients with decompensated cirrhosis (DC). A total of 623 DC patients were recruited from a retrospective observational cohort study. They were admitted to our hospital from January 2014 to December 2015. NAR of each patient was calculated and analyzed for the association with 90-day liver transplantation-free (LT-free) outcome. The performance of NAR and the integrated model were tested by a receiver-operator curve (ROC) and C-index. The 90-day LT-free mortality of patients with DC was 10.6%. NAR was significantly higher in 90-day non-survivors than in survivors (The median: 1.73 vs 0.76, *P* < 0.001). A threshold of 1.40 of NAR differentiated patients with a high risk of death (27.45%) from those with a low risk (5.11%). By multivariate analysis, high NAR was independently associated with poor short-term prognosis (high group: 5.07 (2.78, 9.22)). NAR alone had an area under the ROC curve of 0.794 and C-index of 0.7789 (0.7287, 0.8291) in predicting 90-day mortality. The integrated MELD–NAR (iMELD) model had a higher area under the ROC (0.872) and C-index (0.8558 (0.8122, 0.8994)) than the original MELD in predicting 90-day mortality. NAR can be used as an independent predictor of poor outcomes for patients with DC during short-term follow-up.

## Introduction

Liver cirrhosis is one of the major disease burdens worldwide. In the last decade, the natural course of cirrhosis has been further understood and two distinct stages of the disease have been identified: compensated and decompensated cirrhosis (DC)^1–6^. Decompensated cirrhosis, causing about 1 million deaths annually, ranks 14th among the leading causes of death in adults^7^. It is estimated that 1.5–5% of patients progress from compensated cirrhosis to decompensated cirrhosis annually, with a 5-year mortality of 85% without liver transplantation^8^. For patients with end-stage liver cirrhosis, liver transplantation (LT) remains the only treatment option^9^. However, LT was limited due to the lack of liver donors, death on the waiting list and high medical expenses^10^. Thus, it is of paramount significance to identify DC patients who would benefit from early LT in clinical practice.

The main non-invasive methods used to assess the prognosis of cirrhosis at this stage are the Model for End-Stage Liver Disease (MELD) and Child–Pugh scores. But the application of Child–Pugh score is limited by the fact that the scoring criteria contain some subjective indicators. In contrast, the MELD score is more reproducible and accurate than the Child–Pugh score^11^. Hence, it is necessary to find and develop simple, reproducible and non-invasive indicators to predict the prognosis of patients with LC.

Systemic inflammation (SI) has been identified as a key pathogenic mechanism exacerbating the short-term prognosis of DC^12^. A variety of serum-based indicators of SI have been explored to assess the potential prognostic utility in patients with DC. For example, international normalized ratio to albumin ratio^13^, hemoglobin-to-red cell distribution width ratio^14^, and C-reactive protein-to-albumin ratio^15^ were proven to be used for disease severity stratification and prognosis prediction in patients with DC. Neutrophil count to albumin ratio (NAR) has been identified as a novel inflammatory biomarker predicting prognosis in patients with end-stage pancreatic cancer, rectal cancer and COVID-19^16–18^.

However, the influence of NAR on the prognosis of patients with decompensated cirrhosis remains to be established. Therefore, this study aimed to investigate the prognostic value of NAR in predicting short-term mortality of patients with decompensated cirrhosis.

## Methods and materials

### Data source

We retrospectively investigated data from DC patients aged ≥ 18 years who were admitted to The First Affiliated Hospital, Zhejiang University School of Medicine from January 2014 to December 2015. For patients with recurrent hospitalizations, we only included the first admission.

The exclusion criteria were as follows: (1) age less than 18 or greater than 80 years; (2) hospitalization equal or longer than 2 weeks; (3) all malignancies including hepatocellular carcinoma (HCC); (4) receiving liver transplantation; (5) human immunodeficiency virus (HIV) infection; (6) receiving any type of immunosuppressant medication (including steroids and other immunosuppressive agents) within 1 month prior to admission. Cirrhosis was diagnosed in patients with chronic liver disease who have endoscopic signs of portal hypertension, radiological evidence of liver nodularity, or clinical evidence of previous hepatic decompensation (including hepatic encephalopathy, ascites, and upper gastrointestinal bleeding). Decompensated cirrhosis is defined as the progression of cirrhosis beyond the compensatory capacity of liver function, with symptoms such as portal hypertension, ascites, hepatic encephalopathy, or upper gastrointestinal bleeding. Acute decompensated (AD) was defined by the development of at least one among complications such as ascites, variceal bleeding, hepatic encephalopathy, or non-obstructive jaundice (≥ 5 mg/dL) in the recent 2 weeks^19^. Written consent for our study was obtained from each participant or their legal representative. The study was conducted in accordance with the Declaration of Helsinki, and approved by the Ethics Committee of the First Affiliated Hospital, School of Medicine, Zhejiang University (2015157). All participants or legal representatives provided written informed consent prior to enrolment in the study.

### Patient management

Six hundred and twenty-three patients met the inclusion/exclusion criteria. Management of complications of different etiologies for cirrhosis is in accordance with current international guidelines^1,20^.

### Data collection and follow-up

The following demographic information and laboratory data were collected at the time of admission: age, sex, causes of cirrhosis, acute decompensation, c-reactive protein [CRP], total bilirubin [TB], albumin, white blood cell [WBC], neutrophil, creatinine [Cr], aspartate aminotransferase [AST], alanine aminotransferase [ALT], international normalized ratio [INR], hemoglobin, platelets [PLT] and serum sodium. All patients were followed up for at least 90 days to identify their 90-day survival status, which was verified by medical records, telephone contact, or visiting.

### Assessment of NAR

NAR was defined as neutrophil (10^9^/L) divided by albumin (g/dL). Neutrophil was analyzed by the automatic flow cytometer, while albumin level was generated by the biochemical analyzer.

### Statistical analysis

All continuous variables were expressed as mean ± SD or median (IQR), and categorical data were calculated as percentages. Differences between variables were weighted using analysis of the Student *t*-test or Mann–Whitney *U*-test, respectively. Categorical data were evaluated by the *χ*^2^ test or Fisher’s exact test.

Cox proportional hazards models were used to examine the relationship between NAR and 90-day adverse outcomes.

The results were presented as Hazard ratio (HR) with 95% confidence interval (CI). In multivariate analyses, we computed one unadjusted and two adjusted models. The confounders selected in our models were based on their associations with the outcomes of interest or a change in effect estimate of more than 10%. In the model I, we adjusted covariates for sex, jaundice, HBV, ALT and TB. In model II, covariates were adjusted further for sex, Hepatic encephalopathy, ascites, jaundice, HBV, alcohol, CRP, PLT, ALT, AST, serum sodium, TB and INR. We then applied a generalized additive model to estimate the independent relationship between NAR and 90-day mortality, adjusted for potential confounders (Fig. 2). We further applied a two-piece-wise linear regression model to test the threshold effect of NAR on 90-day mortality according to the smoothing plot (Table 4).

In addition, we performed stratification analysis on causes of cirrhosis and decompensation events to confirm whether the effect of NAR differs in each of the subgroups.

Area under the receiver operating characteristic curve (AUROC) was measured to estimate and compare the discrimination abilities of NAR and MELD score. The areas under the curve (AUCs) of the scoring systems were compared using the DeLong test^21^. The optimal cut-off values for ROC curves were established using the Youden index.

The statistical analyses were carried out using SPSS 26.0, EmpowerStats V.4.1 (http://www.empow-erstats.com/cn/) and R software V.4.2.0 for all statistical analysis. For all statistical analyses, *P* < 0.05 was considered to indicate statistical significance.

The NAR scores were regrouped into two categories (relatively low risk and high risk) using X-tile software (Version 3.6.1, Yale University, USA) to calculate the optimal cut-off values.

The statistical principle behind X-tile softwared is "enumeration method", that is, statistical test is carried out by grouping different values as cut-off values. The result with the smallest *P*-value of test result can be regarded as the best cut-off value, that is, the result of learning from data.

## Results

### Study population

**Figure 1:**
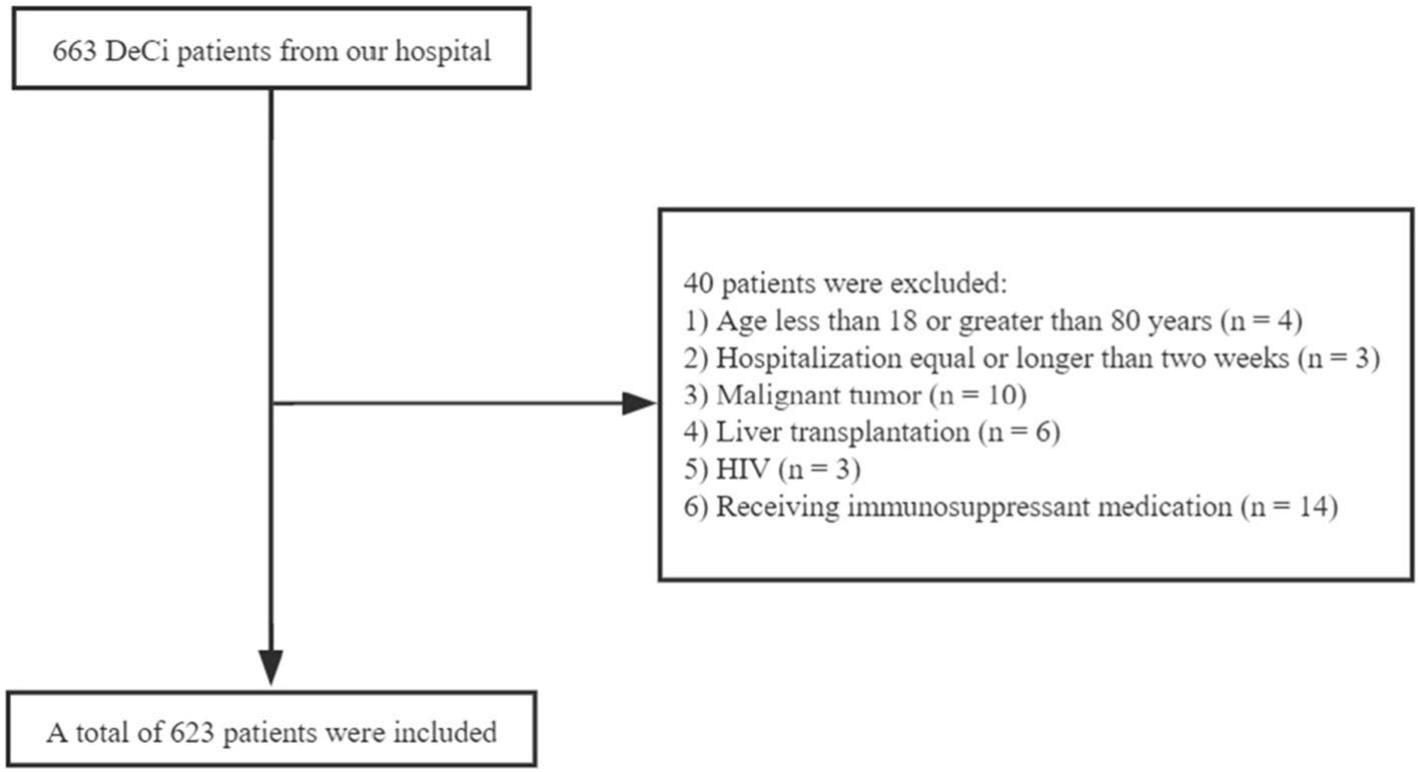
Flow diagram of the participants enrolled.

Finally, 623 patients with acute decompensated cirrhosis were enrolled (470 males, age ± SD: 54.6 ± 11.6) (Fig. 1). As shown in Table 1, the etiology of cirrhosis was hepatitis B virus (HBV) in 414 (66.5%), alcohol related in 92 (14.8%), mixed (alcohol plus HBV) in 6 (1.0%), and of other origins in the remaining 117 (18.8%). The decompensation events for hospitalization were ascites in 199 patients (31.9%), jaundice in 100 patients (16.1%), gastrointestinal bleeding in 99 patients (15.9%), infection in 91 patients (14.6%) and hepatic encephalopathy in 30 patients (4.8%).

**Table 1:** Patient characteristics at baseline.

Variables	Overall (n = 623)	Survivors (n = 557)	Nonsurvivors (n = 66)	*P* value
Demographic				
Age (years)	54.6 ± 11.6	54.6 ± 11.3	55.4 ± 13.8	0.649
Male (n, %)	470 (75.4)	425 (76.3)	45 (68.2)	0.147
Causes of cirrhosis (n,%)				
HBV	414 (66.5)	364 (65.4)	50 (75.8)	0.090
Alcohol	92 (14.8)	88 (15.8)	4 (6.1)	0.035
HBV plus Alcohol	6 (1.0)	6 (1.1)	0 (0)	1.000
Others	117 (18.8)	105 (18.9)	12 (18.2)	0.083
AD (n, %)				
Ascites	199 (31.9)	169 (30.3)	30 (45.5)	0.013
Gastrointestinal bleeding	99 (15.9)	91 (16.3)	8 (12.1)	0.376
Jaundice	100 (16.1)	63 (11.3)	37 (56.1)	< 0.001
Hepatic encephalopathy	30 (4.8)	23 (4.1)	7 (10.6)	0.043
Laboratory parameters				
CRP (mg/L)	7.9 (14.2)	7.5 (13)	12.3 (19)	< 0.001
ALT (IU/L)	27 (32)	26 (30)	56 (87)	< 0.001
AST(IU/L)	45 (56)	45 (52)	95 (121)	< 0.001
TB (μmol/L)	33.0 (71.0)	34.0 (59.5)	225 (367.5)	< 0.001
Hemoglobin (g/L)	101 ± 25	101 ± 25	105 ± 23	0.335
INR	1.3 (0.3)	1.3 (0.4)	1.7 (1.0)	< 0.001
WBC (10^9^/L)	4.0 (3.9)	3.7 (3.5)	8.1 (6.2)	< 0.001
Neutrophil (10^9^/L)	2.4 (2.6)	2.3 (2.5)	5.7 (5.9)	< 0.001
Albumin (g/dL)	3.0 ± 0.6	2.9 ± 0.6	2.9 ± 0.5	0.234
NAR	0.79 (0.92)	0.76 (0.87)	1.73 (2.28)	< 0.001
Cr (μmol/L)	70 (29)	70 (30)	73 (39)	0.074
PLT (10^9^/L)	69 (66)	67 (70)	80 (95)	0.156
Serum sodium (mmol/L)	140 (5)	140 (5)	138 (6)	< 0.001
MELD score	9.8 (9.6)	9.8 (9.0)	20.9 (13.0)	< 0.001

Data was expressed by mean ± standard deviation (SD), median (IQR) or number (percent) and comparisons between variables were performed by the Mann—Whitney *U* test, Student *t* test and a Chi-square test.

*AD* acute decompensation, *ALT* alanine transaminase, *AST* aspartate aminotransferase, *CRP* c-reactive protein, *Cr* creatinine, *HBV* hepatitis B virus, *INR* international normalized ratio, *MELD* model for end-stage liver disease, *PLT* platelets, *TB* total bilirubin, *WBC* white blood cell, *NAR* neutrophil count to albumin ratio.

A total of 66 patients (10.6%) died within 90 days. Of note, none of the patients received a liver transplant during the entire 90-day follow-up period. The death group had significantly higher incidence of ascites, jaundice, and higher CRP, ALT, AST, TB, INR, WBC, Neutrophil, NAR, Serum sodium and MELD score than the survival group.

### Association of NAR with 90-day mortality

The median NAR ratios were 0.76 (0.87) and 1.73 (2.28) for the survival and death groups, respectively (*P* < 0.001) (Table 1). The X-Tile software was used to classify patients into low-risk (NAR < 1.40) and high-risk (NAR ≥ 1.40) groups with 90-day mortality rates of 5.11% (24/470) and 27.45% (42/153), respectively (Table 2).

**Table 2:** Clinical data according to NAR values.

Variables	Overall (n = 623)	NAR < 1.40 (n = 470)	NAR ≥ 1.40 (n = 153)	*P* value
Demographic				
Age (years)	54.6 ± 11.6	54.7 ± 11.4	54.5 ± 12.2	0.973
Male (n, %)	470 (75.4)	344 (73.2)	126 (82.4)	0.022
Causes of cirrhosis (n, %)	414 (66.5)	312 (66.4)	102 (66.7)	0.949
HBV				
Alcohol	92 (14.8)	68 (14.5)	24 (15.7)	0.712
HBV plus alcohol	6 (1.0)	4 (0.9)	2 (1.3)	0.980
Others	117 (18.8)	90 (19.1)	27 (17.6)	0.679
AD (n, %)				
Infection	91 (14.6)	56 (11.9)	35 (22.9)	0.001
Ascites	199 (31.9)	136 (28.9)	63 (41.2)	0.005
Gastrointestinal bleeding	99 (15.9)	71 (15.1)	28 (18.3)	0.348
Jaundice	100 (16.1)	52 (11.1)	48 (31.4)	< 0.001
Hepatic encephalopathy	30 (4.8)	19 (4.0)	11 (7.2)	0.114
Laboratory parameters				
CRP (mg/L)	7.9 (3.0–17.2)	6.2 (2.6–12.8)	15.8 (7.6–29.1)	< 0.001
ALT (IU/L)	27 (18–50)	25 (17–45)	37 (21–86)	< 0.001
AST(IU/L)	45 (29–85)	40 (28–75)	65 (36–127)	< 0.001
TB (μmol/L)	33.0 (19.0–90.0)	30.0 (18.0–59.0)	79.5 (23.0–275.8)	< 0.001
Hemoglobin (g/L)	101 ± 25	99 ± 25	105 ± 25	0.016
INR	1.3 (1.2–1.5)	1.3 (1.1–1.5)	1.4 (1.2–1.8)	< 0.001
WBC (10^9^/L)	4.0 (2.6–6.5)	3.3 (2.3–4.6)	8.7 (6.7–11.5)	< 0.001
Neutrophil (10^9^/L)	2.4 (1.5–4.1)	1.8 (1.2–2.7)	6.2 (4.8–9.0)	< 0.001
Albumin (g/dL)	3.0 ± 0.6	3.0 ± 0.6	2.8 ± 0.6	< 0.001
NAR	0.79(0.48–1.41)	0.64(0.40–0.95)	2.21(1.70–3.18)	< 0.001
Cr (μmol/L)	70 (58–87)	69 (58–85)	72 (61–98)	0.015
PLT (10^9^/L)	69 (43–109)	63 (41–97)	97 (58–153)	< 0.001
Serum sodium (mmol/L)	140 (137–142)	140 (138–142)	137 (133–140)	< 0.001
MELD score	9.8 (5.8–15.4)	9.1 (5.2–13.4)	14.3 (7.8–21.1)	< 0.001
90-day mortality (n, %)	66 (10.6)	24 (5.1)	42 (27.5)	< 0.001

*AD* acute decompensation, *ALT* alanine transaminase, *AST* aspartate aminotransferase, *CRP* c-reactive protein, *Cr* creatinine, *HBV* hepatitis B virus, *INR* international normalized ratio, *MELD* model for end-stage liver disease, *PLT* platelets, *TB* total bilirubin, *WBC* white blood cell, *NAR* neutrophil count to albumin ratio. Data was expressed by mean ± standard deviation (SD), median (P25, P75) or number (percent) and comparisons between variables were performed by the Mann—Whitney *U* test, Student *t* test and a Chi-square test.

### Clinical and laboratory findings associated with NAR Levels

The optimal cut-off levels of NAR were 1.40. Baseline demographic and clinical characteristics stratified by NAR are summarized in Table 2. The participants were divided into two groups based on the cutoff value of NAR: a low group (NAR < 1.40) and a high group (NAR ≥ 1.40). Patients with NAR ≥ 1.40 had higher mortality than those with NAR < 1.40. The NAR ≥ 1.40 group had a significantly higher proportion of male, a higher incidence of infection, ascites, jaundice, and higher CRP, ALT, AST, TB, INR, WBC, Neutrophil, NAR, Cr, PLT, and MELD scores, but lower serum sodium and albumin than the NAR < 1.40 group (Table 2).

### NAR independently associated with 90-day mortality

As shown in Table 3, multivariate analysis demonstrated that NAR remained an independent factor of the 90-day outcome after adjustment for other confounders. We found that a higher NAR was associated with a higher risk of death for the primary outcome of 90-day mortality. For the sensitivity analysis, we considered the NAR as a categorical variable (binary group) and observed the same trend. The HR (95% CI) values of the high group (NAR ≥ 1.40) were 6.21 (3.76, 10.25) when compared with the reference (NAR < 1.40). After adjusted for sex, HBV, ALT and TB in model I, an increasing trend was still observed, with the adjusted HR (95% CI) values of 4.99 (2.93, 8.53) for 90-day mortality at NAR ≥ 1.40. After adjusted for possible confounding risk factors in model II, the upward trend is still statistically significant. (High group: 5.07 (2.78, 9.22)).

**Table 3:** Relationship between NAR and 90-day mortality.

	Crude model		Model I		Model II	
	HR (95% CI)	*P*-value	HR (95% CI)	*P*-value	HR (95% CI)	*P*-value
NAR as continuous						
NAR	1.94 (1.66, 2.26)	< 0.001	1.78 (1.51, 2.10)	< 0.001	2.04 (1.64, 2.53)	< 0.001
Sex						
Male	Refrence		Refrence		Refrence	
Female	1.48 (0.88, 2.48)	0.141	2.97 (1.70, 5.20)	0.001	2.55(1.38, 4.71)	0.003
HBV						
No	Refrence		Refrence		Refrence	
Yes	1.62 (0.92, 2.84)	0.095	2.15 (1.20, 3.86)	0.010	1.52(0.75, 3.07)	0.248
ALT	1.00 (1.00, 1.00)	0.002	1.00 (1.00, 1.00)	0.876	1.00 (1.00, 1.00)	0.442
TB	1.00 (1.00, 1.01)	< 0.001	1.00 (1.00, 1.01)	< 0.001	1.00 (1.00, 1.00)	< 0.001
Hepatic encephalopathy						
No	Refrence				Refrence	
Yes	2.48 (1.13, 5.43)	0.023			1.86 (0.74, 4.67)	0.188
Ascites						
No	Refrence				Refrence	
Yes	1.84 (1.13, 2.98)	0.014			1.87 (1.12, 3.13)	0.016
Alcohol						
Yes	Refrence				Refrence	
No	2.79 (1.02, 7.67)	0.047			4.01 (0.94, 17.16)	0.061
CRP	1.01 (1.01, 1.02)	0.001			1.00 (0.98, 1.01)	0.362
PLT	1.00 (1.00, 1.00)	0.087			1.00 (1.00, 1.00)	0.518
AST	1.00 (1.00, 1.00)	< 0.001			1.00 (1.00, 1.00)	0.277
Serum sodium	0.92 (0.88, 0.97)	0.001			1.01 (0.96, 1.06)	0.759
INR	1.61 (1.44, 1.80)	< 0.001			1.19 (0.98, 1.44)	0.081
NAR quartile						
< 1.40	Refrence		Refrence		Refrence	
≥ 1.40	6.21 (3.76, 10.25)	< 0.001	4.99 (2.93, 8.53)	< 0.001	5.07 (2.78, 9.22)	< 0.001
Sex						
Male	Refrence		Refrence		Refrence	
Female	1.48 (0.88, 2.48)	0.141	3.10 (1.77, 5.45)	< 0.001	2.81 (1.51, 5.24)	0.001
HBV						
No	Refrence		Refrence		Refrence	
Yes	1.62 (0.92, 2.84)	0.095	1.90 (1.06, 3.40)	0.030	1.57(0.77, 3.18)	0.214
ALT	1.00 (1.00, 1.00)	0.002	0.99 (1.00, 1.00)	0.876	1.00 (1.00, 1.00)	0.191
TB	1.00 (1.00, 1.01)	< 0.001	1.00 (1.00, 1.01)	< 0.001	1.00 (1.00, 1.00)	< 0.001
Hepatic encephalopathy						
No	Refrence				Refrence	
Yes	2.48 (1.13, 5.43)	0.023			1.74 (0.67, 4.53)	0.255
Ascites						
No	Refrence				Refrence	
Yes	1.84 (1.13, 2.98)	0.014			1.83 (1.09, 3.08)	0.022
Alcohol						
No	Refrence				Refrence	
Yes	2.79 (1.02, 7.67)	0.047			3.03 (0.76, 12.18)	0.118
CRP	1.01 (1.01, 1.02)	0.001			1.00 (0.99, 1.01)	0.713
PLT	1.00 (1.00, 1.00)	0.087			1.00 (1.00, 1.00)	0.495
AST	1.00 (1.00, 1.00)	< 0.001			1.00 (1.00, 1.00)	0.200
Serum sodium	0.92 (0.88, 0.97)	0.001			1.00 (0.95, 1.04)	0.798
INR	1.61 (1.44, 1.80)	< 0.001			1.23 (1.00, 1.51)	0.046

Crude model: not adjusted for other covariates.

Model I: adjusted for sex, HBV, ALT and TB.

Model II: adjusted for sex, Hepatic encephalopathy, ascites, HBV, alcohol, CRP, PLT, ALT, AST, serum sodium, TB and INR.

*CI* confidence interval, *ALT* alanine transaminase, *AST* aspartate aminotransferase, *CRP* c-reactive protein, *HBV* hepatitis B virus, *INR* International normalized ratio, *PLT* platelets. *TB* total bilirubin, *NAR* neutrophil count to albumin ratio.

### Nonlinear relationship between NAR and 90-day mortality

**Figure 2:**
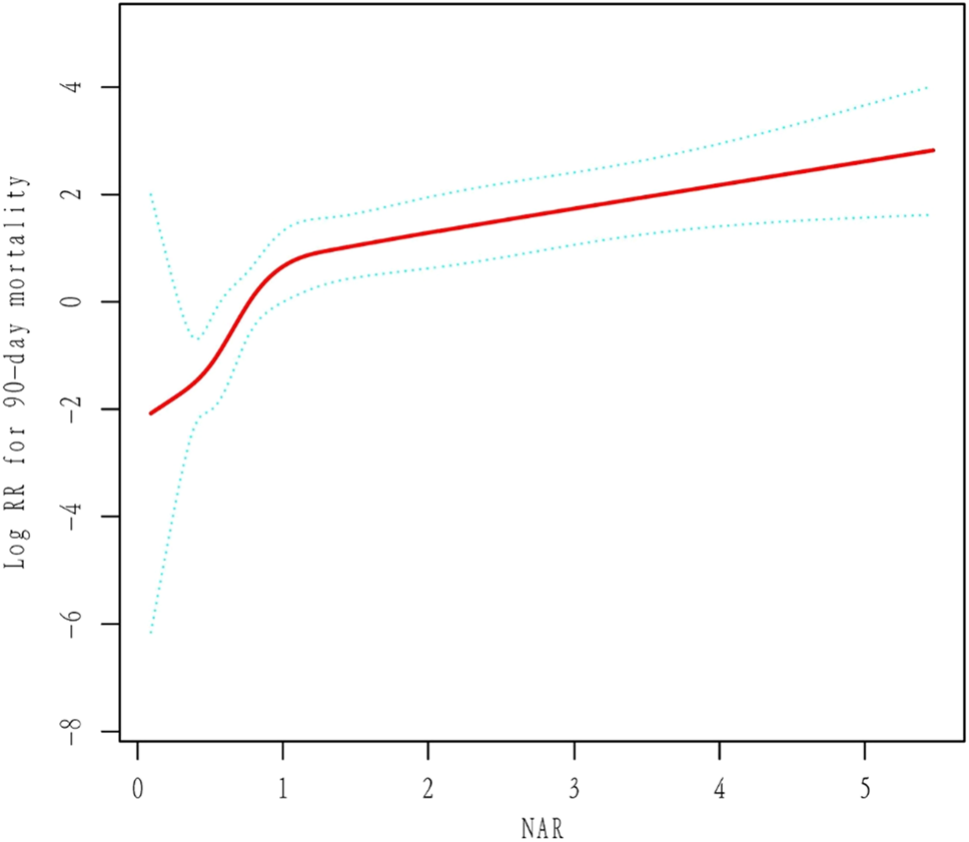
The correlation between NAR and 90 days of mortality. A nonlinear threshold link between NAR and 90-day mortality was found in the general additive model (GAM). The solid rad line shows the balanced curve between the variables. Blue bands show the 95% confidence interval from the adaptation. All adjusted for sex, Hepatic encephalopathy, ascites, HBV, alcohol, CRP, PLT, ALT, AST, serum sodium, TB and INR.

The smoothing plot showed a clear relationship between NAR and 90-day mortality (Fig. 2). Therefore, a threshold analysis was performed using piecewise linear regression, and the results indicated that 0.93 was the optimal inflection point. After adjusting for potential confounders, the threshold effect of NAR on 90-day mortality was significant. The regression coefficient was 74.06 (95% CI 5.34, 1027.98) for Log RR for 90-day mortality (NAR < 0.93) while 1.61 (95% CI 1.27, 2.05) for Log RR for 90-day mortality (NAR ≥ 0.93) (Table 4).

**Table 4:** Threshold effect analysis of NAR and 90-day mortality using piece-wise linear regression.

Inflection Point of NAR	Crude model		Adjusted model	
	HR (95%CI)	*P*-value	HR (95%CI)	*P*-value
< 0.93	75.34 (8.57, 662.45)	< 0.001	74.06 (5.34, 1027.98)	0.001
≥ 0.93	1.55 (1.27, 1.89)	< 0.001	1.61 (1.27, 2.05)	< 0.001

Crude model: not adjusted for other covariates.

Adjusted Model: adjusted for sex, Hepatic encephalopathy, ascites, HBV, alcohol, CRP, PLT, ALT, AST, serum sodium, TB and INR.

*CI* confidence interval, *ALT* alanine transaminase, *AST* aspartate aminotransferase, *CRP* c-reactive protein, *HBV* hepatitis B virus, *INR* International normalized ratio, *PLT* platelets, *TB* total bilirubin, *NAR* neutrophil count to albumin ratio.

### Subgroup analysis

**Figure 3:**
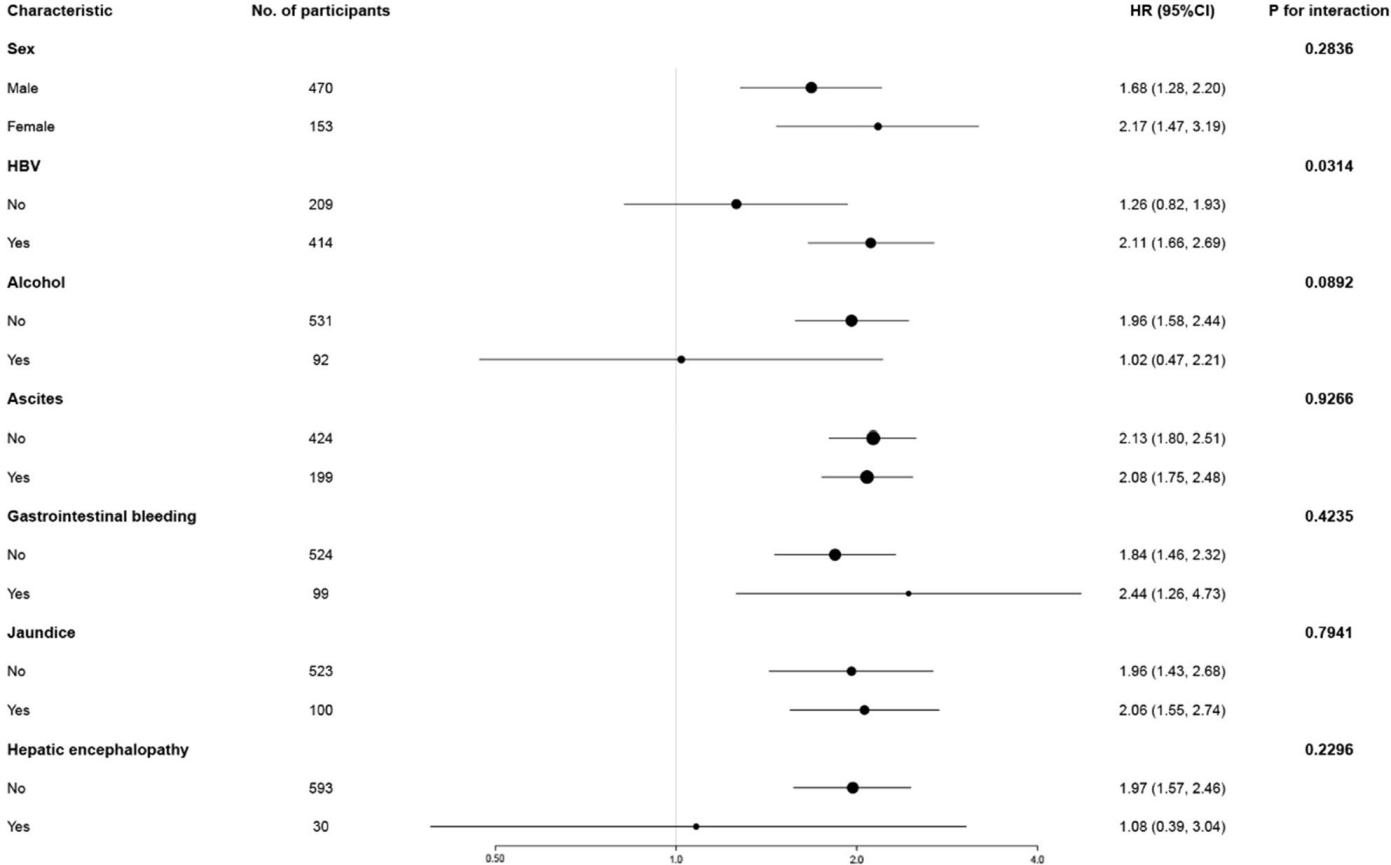
Effect size of NAR on 90-day mortality in prespecified and exploratory subgroups in Each Subgroup. Adjusted for sex, Hepatic encephalopathy, ascites, jaundice, HBV, alcohol, CRP, PLT, ALT, AST, serum sodium, TB and INR except the subgroup variable. *ALT* alanine transaminase, *AST* aspartate aminotransferase, *CRP* c-reactive protein, *HBV* hepatitis B virus, *INR* International normalized ratio, *PLT* platelets, *TB* total bilirubin, *NAR* neutrophil count to albumin ratio.

As shown in Fig. 3, a subgroup analysis of patients with decompensated cirrhosis was performed to determine the concordance between NAR and 90-day mortality. The effect of all subgroup factors on 90-day mortality was not significant, except for the etiology “HBV” (*P* = 0.0314). NAR particularly showed significant interactions in patients with HBV-related decompensated cirrhosis. Patients with HBV had a significant higher 90-day mortality risk (HR (95% CI) 2.11 (1.66, 2.69)) compared to those without (HR (95% CI) 1.26 (0.82, 1.93)).

### The utility of NAR in predicting the 90-day outcome of DC

**Figure 4:**
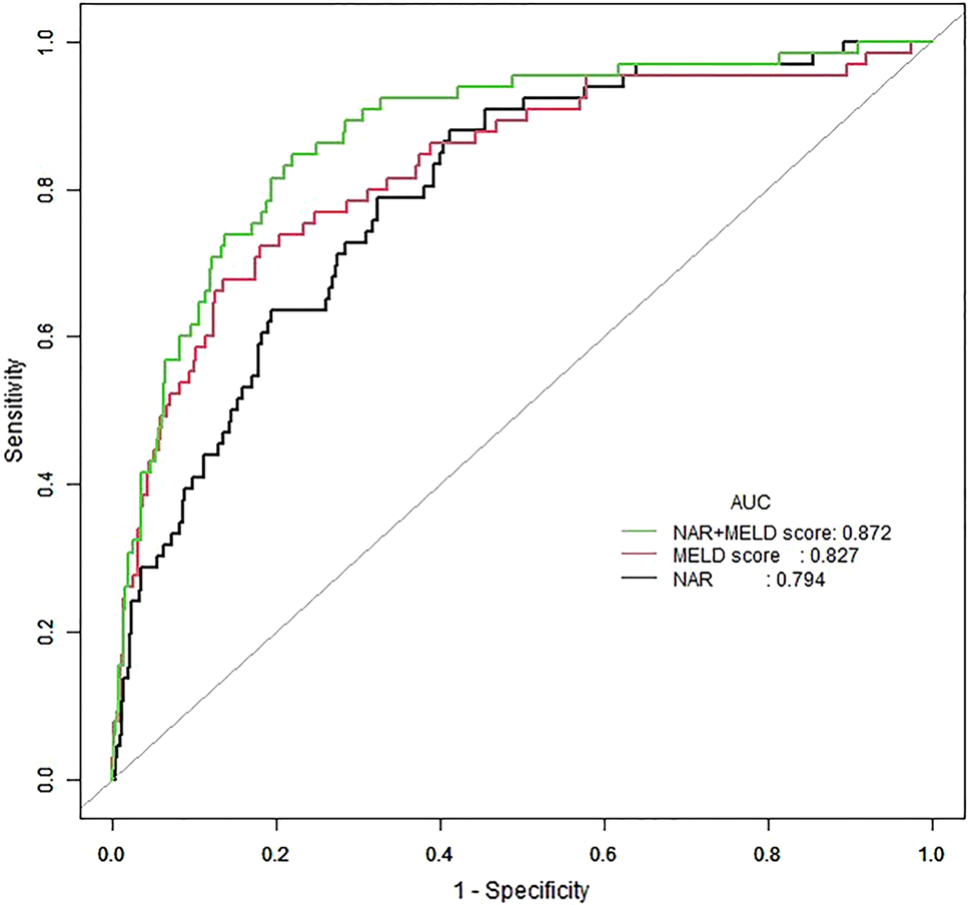
The ROC curve for mortality of 90 days of patients with decompensated cirrhosis. *AUC* area under the curve, *NAR* neutrophil count to albumin ratio, *MELD* model for end-stage liver disease, *ROC* receiver operator characteristic curve.

Receiver operating characteristic (ROC) curve analyses were conducted to assess the abilities of NAR and MELD score to predict poor outcomes in DC patients (Fig. 4). The cutoff values were 15.8 for MELD score (sensitivity, 72.3%; specificity, 81.9%) and 0.87 for NAR (sensitivity, 87.7%; specificity, 58.6%). For the prediction of mortality, the AUC of the NAR was 0.794, slightly lower than the AUC value of the MELD score. (0.827; Z = 0.896; *P* = 0.371). The C statistic for NAR was also lower than the MELD score (0.7789 (0.7287, 0.8291) vs 0.8123 (0.7577, 0.8668)).

### Addition of NAR improved the prognostic accuracy of MELD score

However, when NAR and MELD score were combined, the AUC increased to 0.872. Similarly, the C statistic increased to 0.8558 (0.8122, 0.8994).

### Comparison between NAR and other systemic inflammation-related markers

**Figure 5:**
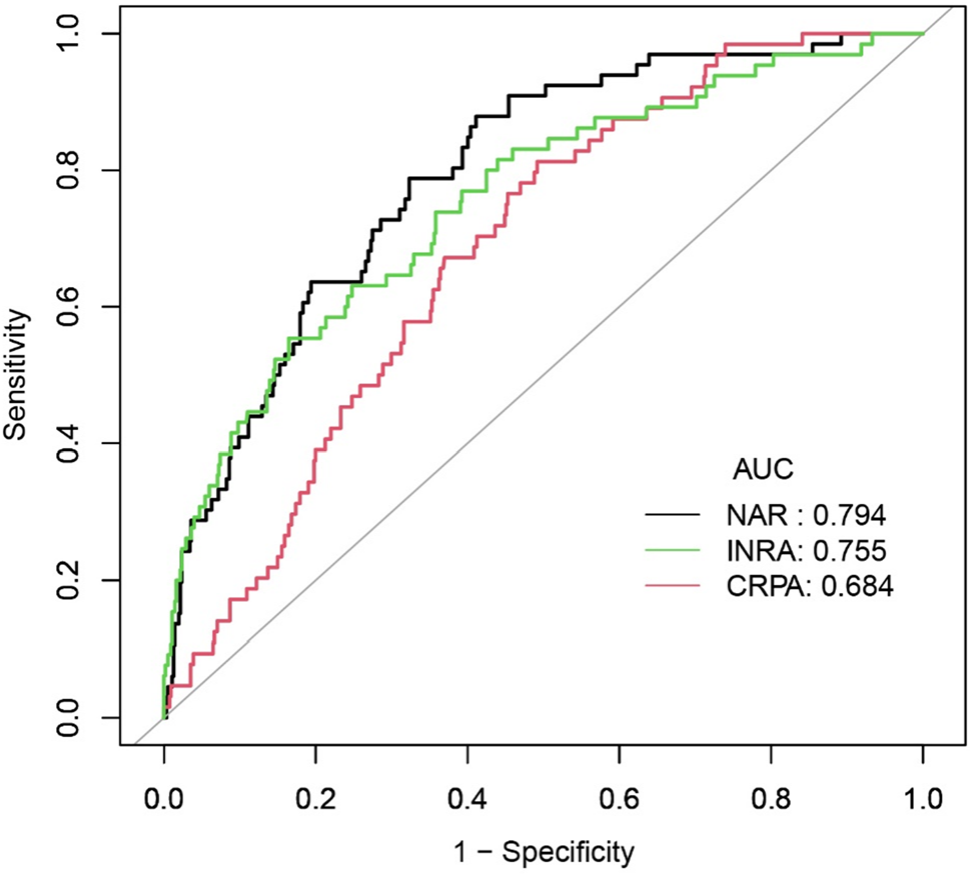
The ROC curve for mortality of 90 days of patients with decompensated cirrhosis by NAR and other parameters of systemic inflammation. *AUC* area under the curve, *NAR* neutrophil count to albumin ratio, *INRA* international normalized ratio to albumin ratio, *CRPA* C-reactive protein-to-albumin ratio, *ROC* receiver operator characteristic curve.

The results of ROC curve analysis (Fig. 5) revealed the area under the curve (AUC) for NAR for predicting mortality to be 0.794, which was superior to both international normalized ratio to albumin ratio (INRA) (0.755) and C-reactive protein-to-albumin ratio (CRPA) (0.684).

## Discussion

In the present study, we found a substantial positive association between NAR and 90-day mortality in patients with DC. Our study showed that elevated NAR was associated with increased 90-day mortality. Multivariate Cox analysis showed that NAR was an independent risk factor predicting adverse outcomes in these patients. Especially in HBV-associated decompensated cirrhosis patients, the effect was particularly obvious. This finding was consistent with that of Han^22^ who found that elevated NAR is associated with poor survival in patients with HBV-DC and has potential clinical application^22^.Recently, the MELD score is the most common scoring system used to classify disease severity and assess prognosis for patients with end-stage liver disease. The MELD score contains three laboratory metrics (total bilirubin, INR and creatinine), which require complex calculations and are not conducive to clinical practice^23^. Both the MELD score and NAR demonstrated comparable predictive power in our study. Moreover, the combination of NAR and MELD can increase prognostic accuracy to 0.872. Therefore, we hypothesized that the relationship between NAR and short-term mortality in DC patients may not be independent, but combined with MELD score. The reason why NAR combined with MELD score is more accurate than NAR and MELD score alone in predicting 90-day mortality in DC patients may be that NAR is introduced into the inflammatory response index based on MELD score. Inflammatory reaction plays an important role in the occurrence and development of liver cirrhosis and affects the prognosis.

There are several possible reasons why NAR could be a new independent risk factor for predicting 90-day mortality in patients with DC.

First, our study revealed that neutrophils were increased in nonsurvivors compared with survivors. Moreover, the neutrophils in the high NAR group were much higher than that in the low group. A critical lot of research supports the significance of systemic inflammation in the development of cirrhosis. Systemic inflammation is related to the disease progression and patient mortality in liver cirrhosis, accompanied by the transition from compensated to decompensated cirrhosis. Systemic inflammatory response syndrome (SIRS) is an exaggerated defense response of the body to a noxious stressor to localize and then eliminate the endogenous or exogenous source of the insult^24,25^. Although the pathophysiology of Acute-on-chronic liver failure (ACLF) remains to be studied. Some studies have shown that SIRS may play an important role in the occurrence and development of ACLF^26–28^. Unlike patients with acute decompensation but no organ failure, patients with ACLF have severe systemic inflammation and oxidative stress. Studies of ACLF have shown that systemic inflammation is directly related to the severity of the syndrome; the greater the intensity of systemic inflammation, the greater the number of organ failures, and the higher the short-term mortality rate^27^. The systemic inflammatory response is often accompanied by excessive release of proinflammatory cytokines. These inflammatory cytokines stimulate and activate neutrophils and promote their phagocytosis and bactericidal efficacy^29^. In return, neutrophils promote inflammation by secreting a range of cytokines such as IL-1 or IL-8 and by releasing a number of enzymes containing particles such as oxidants, proteases and antimicrobial proteins that mediate liver inflammation, apoptosis and hepatocyte necrosis^30^. After the onset of an acute infection, neutrophils are the predominant immune cells driving the early inflammatory response, and neutrophil count can be used as a significant indicator of systemic infection. Therefore, neutrophils can be rapidly recruited in the acute inflammatory response and can be used as a marker of tissue inflammation and disease severity^30^.

In addition, this study also found that in our cohort, although there was no significant difference in serum albumin between 90-day dead patients and surviving patients, after stratifying NAR, it was found that the serum albumin level in the high NAR group was significantly lower than that in the low NAR group. Serum albumin levels represent systemic inflammation because albumin synthesis decreases with the response to the inflammatory cytokine IL-6^31,32^. Some studies revealed that decreased albumin was related to adverse outcomes in acutely ill patients^33–35^. In addition to inflammatory status, serum albumin has been reported to reflect an individual’s nutrition status^36,37^. Albumin is produced in the liver, and hypoalbuminemia is a common complication in patients with cirrhosis and can lead to ascites or edema, leading to increased mortality^38,39^. Our findings suggest that the increased NAR is mainly caused by increased neutrophils and decreased albumin. Therefore, in our opinion, the predictive value of NAR for short-term mortality in patients with cirrhosis may be related to inflammatory response and liver insufficiency. From what has been discussed above, we believe that NAR is valuable for predicting the prognosis of patients with DC.

It is worth mentioning that in this study, we found that the high NAR group had significantly higher proportion of male, a higher incidence of infection, ascites, jaundice, and higher CRP, ALT, AST, TB, INR, WBC, Neutrophil, Cr, PLT, and MELD scores, but lower serum sodium than the low NAR group. This suggests that gender may be a factor in prognosis, although there is a bias here, since men make up about three-quarters of our total study population. Second, a higher rate of cirrhosis complications was associated with an increased risk of death, which was also consistent with previous studies. Furthermore, the association of higher NAR with higher short-term mortality may be related to higher levels of inflammation, poorer systemic organ function, poorer homeostasis, and lower liver compensation in patients with high NAR.

In a subgroup analysis, stratification of patients according to potential confounders revealed a statistically significant interaction of the etiologic factor of hepatitis B cirrhosis. Patients with hepatitis B-related cirrhosis may have a higher 90-day mortality rate. This may be due to the large population base of patients with hepatitis B-related cirrhosis in our study cohort, suggesting that the clinical application of NAR may be more valuable in the context of hepatitis B, which is the main cause of cirrhosis in China. However, the true mechanism remains unclear.

One interesting finding was that the risk of death increased rapidly with increasing NAR at NAR < 0.93, while the risk of death increased more slowly at NAR ≥ 0.93. Why this difference occurs is not well understood, and more research may be needed to explore its clinical significance.

It is worth exploring that the AUC of NAR is higher than that of INRA and CRPA, which may be because the difference between follow-up CRP level and initial CRP level better reflects its correlation with short-term mortality^40^. Similarly, INR is likely to be the same. We estimate that if the follow-up NAR level can be obtained, its prediction accuracy may be further improved.

In this study, we explored for the first time the relationship between NAR and the short-term prognosis of patients with cirrhosis in the decompensated phase. Inevitably, our research had some limitations. First, this is a retrospective cohort study in a single center. Retrospective studies have their own drawbacks and our study is subject to selection bias. Therefore, a further multicenter prospective study is necessary. Second, the actual inflammatory status of a participant may not be measured by the level of NAR alone. Thus, it is advisable to evaluate systemic inflammatory markers or pro-inflammatory cytokines such as tumor necrosis factor-alpha and interleukin 6, which may help elucidate the mechanism of outcome. Third, NAR was only measured at when patients first admitted to our hospital and not serially, this may cause some bias. Therefore, it is not known whether NAR increases progressively as a patient's condition worsens. It is possible that dynamic assessment of NAR levels during hospitalization may reveal different findings. Fourth, unfortunately, our study did not involve data on ACLF and organ failure, which is incomplete to some extent. It is necessary to explore the relevant situation of ACLF subgroups in further in-depth research. Finally, the lack of a validation cohort is one of the limitations of this study. Further validation of the clinical significance of NAR is necessary to determine whether it can be used as a prognostic biomarker in patients with decompensated cirrhosis.

## Conclusions

In summary, NAR is associated with mortality in patients with DC. NAR is a potential biomarker for predicting short-term mortality in these people. As a simple and inexpensive measure, the prognostic value of NAR is similar to the MELD score. Our findings may improve the ability to monitor patients with decompensated cirrhosis. However, our findings need to be corroborated by further studies.

## Data Availability

The original contributions presented in the study are included in the article, further inquiries can be directed to the corresponding authors.
