# Author Correction: Prognostic value of neutrophil count to albumin ratio in patients with decompensated cirrhosis

**DOI:** 10.1038/s41598-023-49813-8

**Published:** 2023-12-18

**Authors:** Junjie Yao, Xianbin Xu, Kai Gong, Huilan Tu, Zhaoyu Xu, Shaoheng Ye, Xia Yu, Yan Lan, Haoda Weng, Yu Shi

**Affiliations:** 1 State Key Laboratory for Diagnosis and Treatment of Infectious Diseases, National Clinical Research Center for Infectious Diseases, Collaborative Innovation Center for Diagnosis and Treatment of Infectious Diseases, The First Affiliated Hospital, Zhejiang University School of Medicine, Hangzhou, 310000 China; 2 Bethune Third Clinical Medical College, Jilin University, Changchun, 132000 Jilin China

Correction to: *Scientific Reports* 10.1038/s41598-023-44842-9, published online 25 November 2023

The Funding section in the original version of this Article was omitted. The Funding section now reads:

“This research was funded by Fundamental Research Funds for the Central Universities (No.226-2023-00127, 2021FZZX001-41), the National Key Research and Development Program of China (No. 2021YFC2301800 and 2022YFC2304501) and Medical Health Science and Technology Project of Zhejiang Provincial Health Commission (No. 2022490480).”

The original Article has been corrected.

